# A unifying mechanism for protein transport through the core bacterial Sec machinery

**DOI:** 10.1098/rsob.230166

**Published:** 2023-08-30

**Authors:** William J. Allen, Ian Collinson

**Affiliations:** School of Biochemistry, University of Bristol, Bristol BS8 1TD, UK

**Keywords:** protein transport, bacterial secretion, Sec machinery, SecYEG, SecA

## Abstract

Encapsulation and compartmentalization are fundamental to the evolution of cellular life, but they also pose a challenge: how to partition the molecules that perform biological functions—the proteins—across impermeable barriers into sub-cellular organelles, and to the outside. The solution lies in the evolution of specialized machines, translocons, found in every biological membrane, which act both as gate and gatekeeper across and into membrane bilayers. Understanding how these translocons operate at the molecular level has been a long-standing ambition of cell biology, and one that is approaching its denouement; particularly in the case of the ubiquitous Sec system. In this review, we highlight the fruits of recent game-changing technical innovations in structural biology, biophysics and biochemistry to present a largely complete mechanism for the bacterial version of the core Sec machinery. We discuss the merits of our model over alternative proposals and identify the remaining open questions. The template laid out by the study of the Sec system will be of immense value for probing the many other translocons found in diverse biological membranes, towards the ultimate goal of altering or impeding their functions for pharmaceutical or biotechnological purposes.

## Graphical abstract

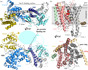

## 1. Introduction

Proteins are synthesized in the cell cytosol, and in the interior of semi-autonomous eukaryotic organelles; those that reside in other cellular compartments, or destined for secretion to the external medium, must therefore cross at least one membrane in order to fulfil their functions. Fifty years ago, César Milstein demonstrated that a secretory protein (an immunoglobulin) was produced as a precursor with a cleavable N-terminal ‘signal’ sequence (SS) accessible at the early stages of protein synthesis [1]. Meanwhile, Günter Blobel had described the propensity of translating ribosomes to interact with membranes of the endoplasmic reticulum (ER) [2]. Blobel then went on to develop the ‘signal hypothesis’, whereby the nascent SS is recognized by a proteinaceous machinery for translocation across the membrane into the ER lumen [3].

In the subsequent years, numerous protein transport machineries have been identified as residents of most biological membranes; both general purpose and highly specific [4–7]. The most prevalent of these is the universal Sec system—the protein translocation channel first postulated by Blobel. The heterotrimeric core Sec translocon is present in every organism in every cell: in the plasma membrane of bacteria and archaea (SecYEG), and the ER membranes of eukaryotes (Sec61). The Sec system turns out to be the primary pathway both for transport across and into (insertion) these membranes [8,9]. Transport into the ER lumen leads subsequently, through vesicular trafficking [10], to secretion to the extracellular medium. Passage across the bacterial plasma (inner) membrane brings about secretion to the cell envelope, including the periplasm and outer membrane of Gram-negatives [11], or to the outside of the cell. Most of what we know about protein secretion is from studies of the Sec machinery of Gram-negative bacteria, from the cytosol across the inner membrane into the periplasm, which will be the focus of this review.

Sec-mediated insertion of a membrane proteins generally occurs concomitant with their emergence from the ribosome exit tunnel—the co-translational pathway. Transport is driven primarily by favourable insertion of hydrophobic trans-membrane helices (TMHs) into the membrane [12,13]. The tight coupling between translation and translocation limits unnecessary exposure of TMHs to the cytosolic environment, where they might aggregate. In eukaryotes, protein secretion is also mostly co-translational (as originally described by Blobel) [14]. By contrast, secretion in bacteria is usually, but not exclusively, post-translational, i.e. not coupled to protein synthesis [15–18]. However, even ‘post-translational’ transport can start before translation is complete [19], and indeed the early targeting steps are probably initiated co-translationally [20].

From a functional perspective, the use of the post-translational pathway is most likely to be due to transport being intrinsically much faster than translation [21]. There are a limited number of SecYEG sites (approx. 500 per cell), far fewer than there are ribosomes, and upwards of 20% of all protein biomass synthesized in an *Escherichia coli* cell is destined for the cell envelope. Therefore, there would simply not be sufficient time to export the excess ribosomal output if secretion were limited by the rate of translation [21,22]. A fast post-translation mechanism thus developed to deliver the required vast quantities of protein across the inner membrane, and prevent their toxic build up and aggregation in the cytosol.

The post-translational activity of the *E. coli* Sec system can be reconstituted *in vitro* using only the core components: the cytosolic ATPase SecA and ATP are alone sufficient to mediate import of a purified, unfolded model pre-protein (i.e. a secretory protein with its SS present) into lipid vesicles reconstituted with SecYEG (proteo-liposomes, PLs) [8]. Transport through this minimal machinery is relatively slow *in vitro* [22]; many other factors accelerate the reaction *in vivo*, notably the electrochemical gradient across the plasma membrane—the proton-motive force (PMF) [8,23]. Nevertheless, the relative simplicity of the core SecYEG–SecA pathway, along with its fundamental biological importance, have led to a thorough characterization.

Decades of genetic, biochemical, structural and biophysical studies have yielded a detailed understanding of many aspects of the transport process; yet despite this, there is no consensus on the underlying molecular mechanism of transport. This is in large part because of the challenge in correlating movements of the pre-protein through the channel with specific conformational changes induced by the hydrolytic cycle of ATP within SecA. Indeed, it has only recently become possible, without perturbing the system, to measure transport with a time resolution faster than the transport process itself—enabled by the development of a non-invasive assay based on a split version of NanoLuc luciferase [24,25].

We present here our current best understanding of the mechanism of post-translational protein transport in bacteria, and describe how new experimental tools have been instrumental in building this model. These approaches—developed and validated for study of the Sec system—will be invaluable for studying other, less experimentally amenable transport systems, such as the machineries that mediate protein import into eukaryotic organelles [26]. The focus of this review is on the mechanism of secretion itself, here defined as transport across the plasma membrane; for discussion of how the SecYEG interactome coordinates cell envelope biogenesis more holistically, we refer the reader to other recent reviews [11,27].

## 2. To begin at the beginning: targeting of pre-proteins to the Sec machinery

The recognition and targeting of a pre-protein begins very early during its synthesis, with an array of different targeting factors that cluster around the ribosome exit tunnel and interact with the N-terminus as it emerges, as first proposed by Milstein. Strongly hydrophobic leader sequences, usually trans-membrane helices, are generally recruited by the ubiquitous signal recognition particle (SRP), which directs the translating ribosome to SecYEG for co-translational insertion into the inner membrane [17,28–31]. The more moderately hydrophobic sequences characteristic of cleavable SSs of post-translational secretory substrates are also recognized co-translationally—most commonly by the chaperone trigger factor (TF) [28,32,33]. This keeps pre-proteins in a translocation-competent state, and delays their interaction with SecA until synthesis is complete, promoting fully post-translational transport [28]. A subset of pre-proteins with more hydrophobic SSs are instead recognized by SecA directly [20,34,35]; this is thought to promote co-translational secretion, e.g. for proteins that fold too quickly for completely post-translational transport [35]. Direct association of SecA with ribosomes is also thought to assist in the biogenesis of inner membrane proteins with large periplasmic domains, which require SecA for optimal transport [35,36].

Effective transport through Sec requires that pre-proteins are maintained in an unfolded state [37–39], and aggregation must also be prevented, especially for those with exposed hydrophobic surfaces, such as outer membrane proteins (OMPs). The presence of an SS itself has been shown to slow pre-protein folding [40,41], and it has also been demonstrated that secreted proteins have intrinsically lower folding propensity than their cytoplasmic counterparts [42]. Beyond this, several chaperones also engage with pre-proteins to promote a secretion-competent conformation. TF and SecA both slow pre-protein folding, and a subset of pre-proteins also require the specific secretion chaperone SecB [43,44], which interacts directly with SecA to facilitiate transport. General cellular chaperones such a DnaJ/DnaK and GroEL/GroES have also been implicated in pre-protein maintenance [45,46]. Presumably, the number and variety of different Sec substrates necessitates a range of solutions to a general problem—how to ensure the large amounts of protein destined for the cell envelope reach their correct destination while avoiding mishap: misfolding, premature folding, aggregation, degradation, etc. For a recent, more thorough review of the chaperoning and other quality control processes affecting Sec substrates, see [47].

## 3. Structures of the core components of the Sec system

**Figure 1.**
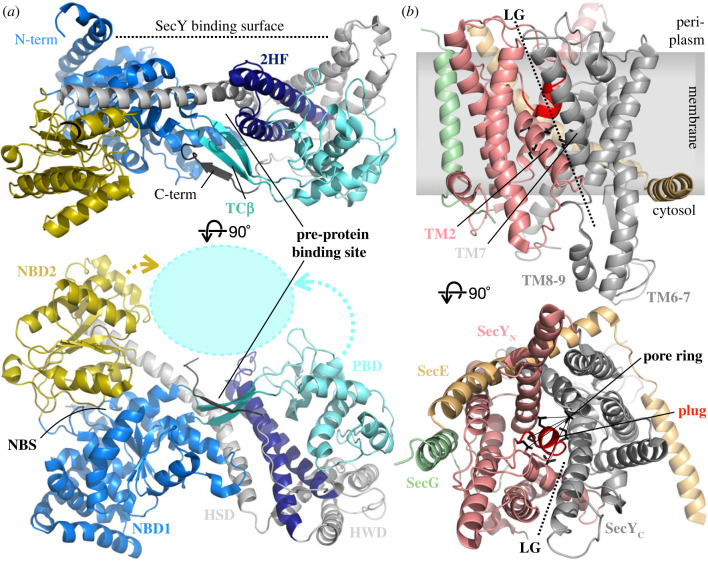
Structures of SecA and SecYEG. (*a*) The first structure of SecA alone (from *Bacillus subtilis*; PDB code 1M6N [48]). Domains and key features are demarcated by colour as indicated (see also text for details), and movements of the PBD and NBD2 in response to SecYEG/pre-protein binding are indicated by dotted arrows. (*b*) The first structure of SecYEG (from *Methanococcus jannaschii*; PDB code 1RHZ [49]). Key features are labelled and distinguished by colour as noted.

The solution of two crystal structures—SecA from *Bacillus subtilis* [48] and SecYEG from the archaeon *Methanococcus janaschii* (alternatively termed SecYE*β*) [49]—were watershed moments for the protein translocation field. The structure of SecA is shown in figure 1*a*: it consists of two RecA-like nucleotide binding domains (NBD1 and NBD2), between which the nucleotide binding site (NBS) is sandwiched; a mobile pre-protein binding domain (PBD, also known as PPXD); and a peripheral helical wing domain (HWD). These domains are arranged around a central helical scaffold (HSD), which also encompasses a two-helix finger (2HF), critical for function. A regulatory C-terminal domain, which can be removed without interfering with the core transport function [50,51], is not resolved in any structure and is most likely mobile.

The central subunit of the protein-channel complex, SecY [49] comprises ten TMHs arranged in two inverted pseudo-symmetric bundles of five between which a pathway is formed through the membrane. This channel is constricted at its centre by a ring of hydrophobic residues (the pore ring) and blocked further on the periplasmic side by a short, moveable α-helical plug domain (figure 1*b*). A lateral gate (LG) separates the channel from the lipid bilayer, with SecE wrapping around the back (the hinge region), maintaining the stability of SecY, while SecG sits peripherally.

The classical view of the bacterial secretory machinery is of SecA as a motor protein, with SecY providing a passive channel in the membrane through which pre-protein translocate. However, it is probably more helpful to view SecA alone more as a chaperone or holdase, with the SecYEG–SecA complex forming the functional transporter. SecA by itself can bind to pre-proteins but is otherwise inactive unless bound to SecYEG; its basal rate of ATPase activity is very low (approx. 0.01 s^−1^) as a result of slow and rate-limiting ADP release [52–54]. SecYEG alone, meanwhile, is closed to prevent leakage of ions across the membrane [49] and has very low affinity for pre-protein [41]. The SecA ATPase activity is strongly stimulated upon binding to SecYEG and acidic phospholipids, with full activation also requiring the presence of pre-protein [54–56]. The conformational changes induced by ATP binding and hydrolysis at the NBS of SecA are then propagated at long range through SecA and SecY, as discussed below.

## 4. Pre-protein recognition by SecA

SecA recognizes pre-proteins destined for secretion in part by their SS—one or more positive charges at the N-terminus, followed by a moderately hydrophobic α-helical region of approximately 8–20 amino acids and then a short stretch of hydrophilic residues containing the cleavage site for signal peptidase [57]. While alone in solution SecA associates with the SS with moderate affinity (low µM K_D_) [58,59]; this interaction affinity is strongly augmented by the presence of exposed hydrophobic patches within the mature domain of unfolded pre-proteins [58,60]. Indeed, SecA will also bind some unfolded pre-proteins even without their signal sequence. These hydrophobic patches act as a secondary targeting factor, since removing them all from a model pre-protein has been shown to compromise its transport by SecA [60].

**Figure 2.**
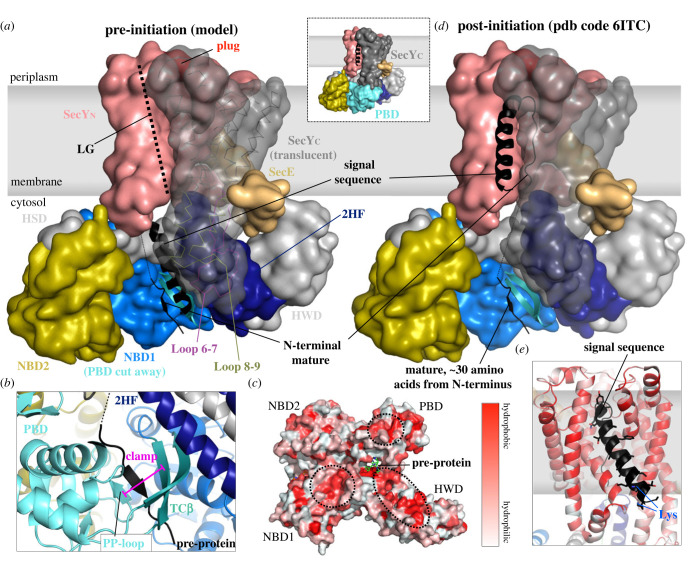
Initiation of protein translocation and pre-protein interactions. (*a*) Surface representation of SecYE-SecA with bound pre-protein before initiation (model based on PDB structure 6ITC [66,67]), coloured as in figure 1. The PBD of SecA has been removed and the C-terminal half of SecY made translucent (with the backbone in ribbon representation), to reveal the inner workings of the complex (compete structure shown as inset). (*b*) Closeup of the SecA clamp; see text for further details. (*c*) Cytosol-facing surface of SecA, coloured by hydrophobicity (red is more hydrophobic), as per [68]. Dashed circles indicate hydrophobic interaction sites for upstream mature regions of the translocating pre-protein [60]. (*d*) As in panel a, but post-initiation (PDB code 6ITC [66]). (*e*) Closeup of the post-initiation LG, coloured by hydrophobicity.

Numerous studies have identified the groove between the HSD and PBD as the binding site for the SS and early mature regions of pre-protein [61–63] (figure 1*a*). Because there is considerable variability in the length and sequence of the pre-protein N-terminal SS, this interaction must by its nature be relatively flexible and non-specific [64]. Although an NMR structure of SecA has been solved with a model signal peptide bound perpendicular to the 2HF [62], MD simulations suggest that its orientation is highly flexible [65]. Meanwhile biochemical and fluorescence resonance energy transfer (FRET) studies suggest that, under physiological conditions, the SS forms an α-helix, which along with the early mature region binds parallel to the 2HF as a hairpin (figure 2*a*) [63,67,69,70].

One early N-terminal section of the mature protein is thought to augment two β-strands that link the PBD and NBD1 (TC*β*, see also below), to form a three stranded β-sheet (figure 2*a,b*) [71,72]. In the absence of pre-protein, this site is occupied by the C-terminus of SecA (figure 1*a*), which probably performs an auto-regulatory role: deletion of this C-terminal region increases the basal ATPase activity of SecA, and its affinity for SS, without affecting transport itself [62,73]. Mutagenesis and cross-linking studies also suggest that regions of the mature polypeptide, C-terminal to this β-strand, interact with hydrophobic patches on the surface of SecA that faces out towards the cytosol (figure 2*c*, dashed circles) [60].

## 5. Association of SecA with SecYEG and membranes

SecA has a strong affinity (approx. 50 nM) for SecYEG under native conditions, i.e. within an acidic phospholipid bilayer [74]. The first structure of the SecYEG–SecA complex [75] revealed the protein–protein contacts involved: most prominently envelopment of the two extended cytoplasmic loop of SecY (between TMHs 6–7 and 8–9) by SecA, and insertion of the 2HF of SecA into the cytoplasmic opening to the SecY channel (figure 2*a*). While these interactions are required for function [76,77], they are not sufficient alone: interactions between SecA and lipids—primarily burial of the SecA N-terminus in the membrane [78]—are also critical. Indeed, SecA binds to lipid bilayers even in the absence of SecYEG, and probably accesses its SecYEG-bound conformation *via* a membrane-associated intermediate [79,80]. Membrane association of SecA is important for its function: transport is prevented by removal of the N-terminus, but can be restored (at least partially) by artificially attaching the truncated N-terminus to the membrane [81].

In solution, SecA self-associates to form a homodimer, but the question of whether this dissociates upon association with the lipid bilayer and/or SecYEG has been the source of many contradictory results (see [82,83] for recent examples). It seems unlikely that dimerization is essential for the translocation process *per se*, given that transport can still occur *in vitro* when SecYEG and SecA are crosslinked together as a 1 : 1 complex [84–86] or as a SecY–SecA fusion [87]. Nevertheless, there is good evidence that two copies of SecA can be present during transport *in vivo* [88,89]. Potentially the second protomer increases transport efficiency and is thus needed *in vivo* to fulfil the demands of envelope biogenesis.

Given that SecA has an affinity for the mature domains of secretory proteins, a plausible explanation for the presence of two protomers during transport is that pre-protein partially mediates SecA self-association. Although only one SecA at a time is actively transporting through SecYEG, a second can be recruited (e.g. to remove residual structure from the upstream mature part of the translocating pre-protein, feeding it to the active translocon in an optimal conformation as transport proceeds). Alternatively, the association of pre-protein bound SecA dimers to SecYEG, and subsequent dissociation of one of the SecAs, could help orient the incoming SS and mature protein for efficient docking and initiation of translocation [86]. Kinetic studies have also shown that the active SecA is able to dissociate from SecYEG during transport, particularly for longer pre-proteins [85,90–92]; so the presence of a second SecA protomer nearby might help re-orient and jump-start the resulting stalled translocon.

## 6. Initiation of transport

The proposed initial binding site for pre-protein on SecA is, at least to some approximation, sterically compatible with the SecYEG–SecA interaction; and indeed positional mapping by FRET suggests that the SS-early mature domain hairpin does not move relative to SecA upon association with SecYEG (in the ADP-bound state; figure 2*a*) [67]. It seems likely, therefore, that the pre-protein can associate with both free SecA or SecA bound to SecYEG. Full initiation—the transition from the structure depicted in figure 2*a* to that in figure 2*d*, in which the SS is inserted into the membrane at the LG of SecY, with its N-terminus facing towards the cytosol and a loop of mature domain threaded through the channel (figure 2*d*)—requires at least ATP binding, and possibly also hydrolysis [23,67,93]. FRET studies on purified systems suggest that turnover is required: ATP-γ-S (a non-hydrolysable, or slowly hydrolysable, ATP analogue) allows the pre-protein to slide further towards the SecY channel but not fully insert [67], while ATP, but not AMPPNP (another non-hydrolysable ATP analogue), allows pre-protein dependent displacement of the plug [93]. In earlier experiments using native *E. coli* membranes, however, AMPPNP binding alone was sufficient for initiation to take place [23].

The exact mechanism of initiation has yet to be established. Kinetics data suggest that initial assembly of the SecYEG–SecA–pre-protein complex (figure 2*a*) is rate-limiting: when all three components are pre-incubated together prior to the addition of ATP, initiation precedes very rapidly [92]. The simplest explanation would appear to be that initiation is just like any other transport step (see below), but highly energetically favourable. The reasoning being that it involves partitioning of the hydrophobic helix of the SS into the membrane–translocon interface at the LG, as well as alignment of the N-terminal positive charge with the negatively charged membrane phospholipid head groups (figure 2*e*).

Several conformational rearrangements occur in the SecY channel in response to SS binding into the LG, which together ‘unlock’ the translocon, activating it for secretion [58,76,94]. These include partial opening of the LG, with TMH7 tilting to accommodate the SS, and displacement of the plug from the periplasmic channel exit site [66,76,93–95]. The requirement for an SS to unlock the channel underscores that there is a trade-off between secreting pre-proteins as fast as possible and avoiding mistargeting of cytosolic proteins. Indeed, several point mutations in Sec components (*prl*, protein localization) are already partially unlocked without the need for an SS [76]; these substantially increase the rate of transport, but also produce a defect in selectivity for *bona fide* secretory pre-proteins [96].

## 7. Interactions between the Sec machinery and pre-protein

Once initiation has taken place, the interactions between the Sec system and pre-protein have been quite clearly captured by structural data (figure 2*d*) [66,95,97]. In SecYEG, the SS is bound tightly in the LG, with the N-terminus tilting outwards and the positive charges contacting the polar headgroups of the phospholipids on the cytoplasmic face of the membrane (figure 2*e*). The mature domain runs as an unfolded polypeptide through the centre of the channel, constricted but making few specific contacts with the channel walls. In all structures, a glycine sits at very centre of the pore ring, snugly enveloped by an annulus of hydrophobic side chains; this suggests that the channel has been trapped in the most closed conformation it can occupy with a pre-protein present, and must open further to allow bulkier amino acids to pass through.

In SecA, the PBD closes around the distal end of the pre-protein, forming a ‘clamp’ that probably holds it in place. The short section of peptide within this clamp is well resolved in the structures where it is present [66,97]: it forms a short β-strand, augmenting TCβ on one side, and with a loop from the PBD (the PP-loop), sandwiching it on the other side to form a small four-strand β-sheet (figure 2*b*).

Closure of the clamp around the pre-protein also has more widespread consequences on the conformation and activity of the translocon. A small movement at one side of the clamp (NBD2) slightly opens the NBS, increasing the rate of ADP release [71,75,98,99]. Additionally, clamp closure is communicated to the interface between SecYEG and SecA, stabilizing the interaction in a nucleotide-dependent manner—preferentially in the ATP-bound state (demonstrated with ATP*γ*S) [81].

The other regions where pre-protein interacts with SecA are poorly resolved in all structures, suggesting that the pre-protein is mobile and not bound in one specific conformation; unsurprisingly so, given the necessary variable and dynamic nature of the interacting channel and pre-protein. Nonetheless, the path of the pre-protein through SecA can easily be inferred from the regions that are resolved (figure 2*d*), and correlates well with the results of earlier cysteine cross-linking studies [100].

The interface between SecY and SecA forms a vestibule that changes shape in response to the nucleotide exchange cycle [101], and within this entrance to the SecY channel pre-protein makes extensive contact with the 2HF—an interaction that is critical for function [102]. Notably, perturbation of the 2HF has a profound effect—increasing the rate of ADP release (the rate-limiting step of the hydrolytic cycle), suggesting it has a role in sensing pre-protein at the channel entrance and coupling it to ATP turnover [84,99].

Given the huge range of possible peptide stretches that can be translocated by the Sec system, the use of β-augmentation at the only fixed point in SecA that grips pre-protein makes sense: it is one of very few protein–protein interactions that is reasonably strong, but only involves atoms in the peptide backbone. Alternatively (or additionally), it has been proposed that small hydrophobic ‘prongs’ in the SecA clamp make transient contact with hydrophobic stretches of pre-protein [98], which would fulfil a similar function, given the high frequency with which hydrophobic residues appear in pre-proteins.

## 8. Conformational changes and the hydrolytic cycle of ATP

**Figure 3.**
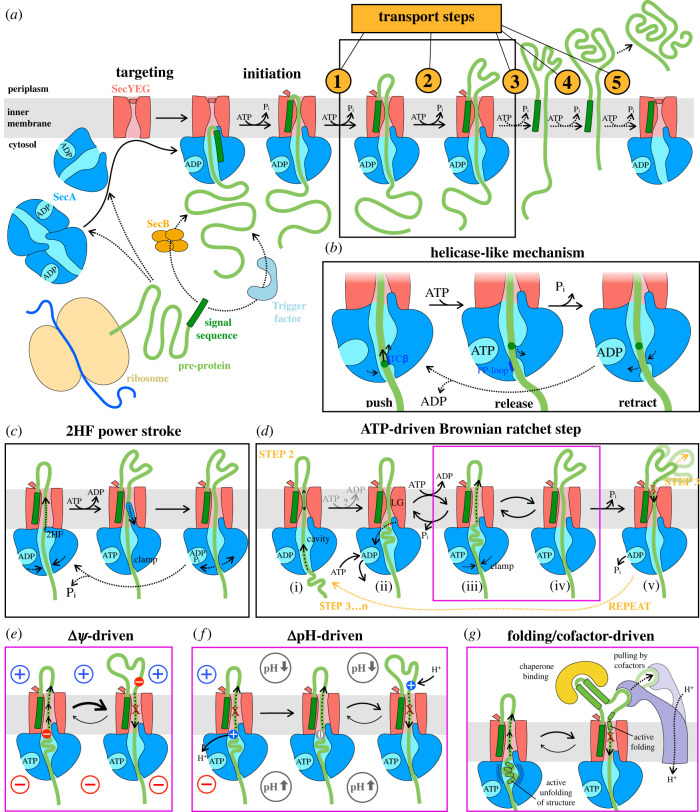
Mechanism of transport. (*a*) Overview of the entire transport process for a pre-protein transported in multiple STEPS (5 as an example; see text for more detail). To reduce figure complexity, the Sec machinery is not shown in later STEPS. The black box highlights a single transport STEP, with proposed mechanisms expanded upon in panels (*b–d*). (*b*) Proposed helicase-like mechanism for ATP-driven transport by SecA, where alternate binding of TCβ and the PP-loop drives transport (see text) [97]. (*c*) Proposed ATP-driven mechanism of transport based on a power stroke by the 2HF [103]. (*d*) Proposed unifying model of ATP-driven transport (detailed in the main text). The entire panel corresponds to a single transport STEP, and is thus one of many repeats (five in this example; orange arrows) to fully transport the protein depicted in panel (*a*). Forward diffusion (magenta box) can be further augmented by other factors, as in panels (*e–g*). (*e–g*) Additional factors that promote forward or prevent reverse polypeptide diffusion during the ATPase cycle: (*e*) electrophoresis of negatively charged residues (Glu and Asp) promotes forward movement and inhibits its reverse; (*f*) ‘proton ratcheting’ of Lys (and potentially His) prevents backsliding; (*g*) regulated unfolding of pre-protein in the cytosol promotes forward movement (left), while refolding in the periplasm, binding of downstream periplasmic factors such as chaperones, or direct pulling as proposed for SecDF all inhibit reverse polypeptide movement, and thereby promote forward translocation (right).

The entire process of pre-protein transport as described so far—targeting, initiation, and stepwise transport of unfolded pre-protein—is depicted schematically in figure 3*a*. At the centre of this schematic lies the big question: what are the individual transport steps? In other words, how is the energy of ATP binding and hydrolysis converted into directional movement? Since the minimal energy requirement of SecYEG–SecA for pre-protein transport is ATP, the conformational changes it undergoes in response to the hydrolytic cycle of ATP must underpin the molecular basis for transport. Under fully translocating conditions, the rates of ADP release and ATP hydrolysis are fairly similar and rate-limiting, with ATP binding and phosphate release much faster: thus the active complex mainly spends its time in the ATP- and ADP-bound forms [54]. Until very recently, the only structures solved of the SecYEG–SecA complex [66,75,95] had ADP•BeF_x_ bound in the NBS, and probably most closely resemble a transient conformation during, or immediately after, ATP hydrolysis. Recently, a structure with ADP was added to this [97]; even in this case, however, ADP•BeF_x_ was required throughout the purification, suggesting that it produces a uniquely stable state—more so than non-hydrolysable ATP analogues.

Various different approaches have been used to convert these trapped snapshots into functional insights. One of these is computational: substitution of the ADP•BeF_x_ in a crystal structure with ATP or ADP followed by MD, then looking for conformational differences [99]—although it should be noted that the time scales accessible to MD are still not long enough to guarantee sampling of all conformational changes.

An alternative approach is to use hydrogen–deuterium exchange (HDX) mass spectrometry, which measures solvent accessibility of the protein backbone with a resolution of short peptides, and thus highlighting structural differences between the ADP- and ATP-bound states [98,101,104]. Likely conformational changes can also be inferred by comparing the various structures of SecYEG and SecA, independently (figure 1) and complexed together (figure 2), and looking for domains that are distinct between them. FRET, furthermore, especially single molecule FRET (smFRET), has proved an effective way to explore the dynamic action of molecular machines: not only is it sensitive to relatively small distance changes between two sites on a protein, but it can also provide detailed kinetics for the transitions between states [93,99,103].

The biggest nucleotide-dependent conformational change is a widening of the channel and adjacent LG in response to ATP (or a non-hydrolysable analogue), and a closure in response to ADP [99]. The conformational changes affecting SecA are somewhat less clear cut: while most evidence suggests that the PBD clamp responds to nucleotide occupancy [71,97,98,103,105–107], it has proved difficult to correlate specific conformations with each of the NBS occupancies. This is also exacerbated by the fact that PBD dynamics appear to depend on the presence of pre-protein, and perhaps even the exact sequence within the clamp [98]. SmFRET experiments using a trapped pre-protein suggest that there is a large-scale closure of the clamp at some point in the ATP turnover cycle, but this closed state is relatively short lived [103]. Furthermore, it is possible that the conformational changes most important for function are more subtle, perhaps revealing and occluding binding sites for pre-protein within the closed clamp.

New structures of the complex with ADP and ADP•BeF_x_, determined by high-resolution cryo-electron microscopy [97], lend support to this notion. The differences between the two structures are relatively subtle and buried within the complex, so not easily probed by FRET. The presence of ADP•BeF_x_ induces a slight closure of the two RecA-like ATPase domains of SecA (NBD1 and NBD2), and this conformation is propagated to the PBD—the pre-protein clamp noted above (figure 2*b*). The short fragment of pre-protein grasped within this region, well resolved thanks to the artificial addition of four consecutive tyrosines, is approximately 4 Å further away from the SecY channel with ADP present, and makes fewer hydrogen bonds with TCβ.

Based on these differences, and by analogy with single-strand DNA/RNA helicases, the authors of this study proposed a ‘hand-switching’ mechanism for protein translocation (figure 3*b*). In this model [97], TCβ binds to the pre-protein—primarily by β-augmentation, but also by some sequence-specific interactions—and then pushes it forwards a short distance, followed by release and retraction. The PP-loop, meanwhile, binds to the pre-protein after the push and holds the pre-protein in place during TCβ retraction.

The homology of SecA with the superfamily 2 helicases has been known for many years; it has even been reported to possess an intrinsic RNA helicase activity [108]. However, mutational analysis shows that loss of the helicase activity does not affect protein transport [109]. Indeed, the residues identified in the new study in contact with the trapped region of the pre-protein are also not necessary for protein transport. It should also be noted that mechanisms of this kind require three distinct states to provide directionality (push, release, retract); and the proposed third state (apo) is an unlikely candidate, as it is only very transiently populated at physiological ATP concentrations [54].

The only other discernible movement in those different EM structures is of the SecA scaffold, which peels slightly away from SecYEG in the ADP state, explaining the lower affinity of SecA_ADP_ for the channel [81,110]. Elsewhere, the structures are remarkably similar. This is puzzling given a number of studies that highlight the dynamic behaviour of the translocon outlined above—particularly the channel component SecYEG—and suggests that some key conformations of this highly flexible complex were not accessible to this particular analysis.

Another point of contention is how the 2HF moves during the ATP hydrolytic cycle. It has been reported that part of SecA—presumably the 2HF—is able to completely cross the membrane, as evidenced by accessibility to crosslinkers or periplasmic protease [111–113]. FRET measurements also show a nucleotide-dependent conformational change in the 2HF of SecA [103]; however, as one of the dyes is tightly constrained it is not possible to determine the nature or distance of this movement. This has led to the proposal of a power-stroke mechanism in which the 2HF pushes the pre-protein into the channel, then resets (figure 3*c*). However, as already noted, the 2HF can be immobilized to the protein channel without preventing transport [84,85]. Moreover, from a purely structural perspective it is hard to envisage the 2HF moving very far into the channel without very large, hitherto unobserved conformational changes. The recent structures of SecYEG and SecA bound to pre-protein also show the 2HF in the same position irrespective of the nucleotide bound.

## 9. The kinetics of pre-protein transport

The mechanism of protein transport cannot be deduced from static structures alone; time-domain information on pre-protein movements through the channel, and how they relate to the kinetics of ATP turnover, are also required. Many studies have determined the average rate of pre-protein transport under different conditions [114–120], but until recently technical limitations have precluded estimation of the kinetic step size (i.e. how many amino acids are transported in a single transport event) [92]. Estimates based on the rates of transport and ATP turnover in bulk give stoichiometries of the order of 5 ATP turnovers to move a single amino acid across the membrane [114]. Protease protection studies, meanwhile, have suggested that a single ATP turnover translocates 1–5 kDa of pre-protein [121,122], which is irreconcilable with the measured ratio of ATP turnover and amino transport rates. Perhaps the latter measure of step size may not be an accurate, as it is subject to other factors such as transport-independent changes in protease protection profile.

These matters were to some extent reconciled with the development of an assay based on a split NanoLuc luciferase [24,25], which has a sensitivity and time-resolution sufficient to allow the testing of specific models [92,123]. Mathematical modelling of NanoLuc traces suggest that transport proceeds in a relatively small number of kinetic steps—roughly five for the model pre-protein pSpy (161 amino acids) [92] (i.e. consistent with the estimates from protease protection). Furthermore, the number and rate of these steps is dependent on amino acid composition of the pre-protein [123]; explaining why the protease protected fragments (representing transport steps) are not evenly spaced on a protein gel, or of equal intensity [23,121,124]. Notably, transport of arginine is by far the slowest of all the amino acids, constituting the majority of total transport time [123].

The small number of kinetic transport steps contrasts sharply with the very large number of ATP turnovers by SecA in the same time period: in effect, each transport step requires something of the order of 100 ATPs *in vitro*—a number that varies depending on pre-sequence or the presence of other factors that slow or accelerate transport. This eliminates any transport model where a single ATP turnover transports a fixed length of pre-protein across the membrane: thus, the ATPase cycle of SecA is only loosely coupled to polypeptide movement through the channel. Moreover, this coupling is very inefficient when only the core Sec components are present.

## 10. A unifying model for ATP-driven translocation

The observation that the primary effect of ATP binding to SecA is opening of the channel through SecY led us to propose a Brownian ratchet mechanism for pre-protein transport [99], shown in modified form in figure 3*d*. Effectively, some parts of the pre-protein diffuse freely though the SecY channel (state (i) in figure 3*d*), but certain ‘difficult’ sequences are unable to cross in the ADP-bound conformation, and trigger nucleotide exchange (state (ii)). ATP binding then opens the channel (state (iii)), allowing these challenging sequences to diffuse through (state (iv)), trapping them on the outside once ATP hydrolysis has occurred (state (v)). The cycle then repeats as shown in figure 3*d* (orange arrows); from STEP 1 to STEP 2, and as required to STEP *n*, around four further times for pSpy, until the C-terminus enters the periplasm.

Subsequent experiments have identified the triggering sequences (at the SecY channel entrance in figure 3*d*(ii)) as positive charges or bulky, hydrophobic patches [123]. We have also shown that ATP binding alters the environment at the interface between SecYEG and SecA to promote secondary structure unfolding in the pre-protein. The environment in this region also causes the deprotonation of lysine residues. Hence, both unfolding and lysine deprotonation (removal of a positive charge) could increase the likelihood of pre-protein diffusion through the channel towards the exterior [101,123] (see also below).

The central contribution of diffusion to this mechanism is appealing in that it sidesteps many of the problems of the other proposed models (figure 3*b,c*)—notably the large and variable number of ATP turnovers per kinetic step. Since ATP turnover merely allows polypeptide movement, rather than physically causing it, not every ATP consumed necessarily gives rise to a productive transport event. However, in its initial form the model is clearly incomplete. If opening of the channel through SecY were the only effect of ATP binding, one would predict that pre-protein can be fully translocated just by adding a non-hydrolysable ATP analogue—at least assuming transport has been initiated first. Yet this is not the case; ATP hydrolysis is also required [23,56,125]. Furthermore, the process of transport is able to unfold some elements of secondary and tertiary structure [39,126,127], with kinetics that suggest active unfolding, as opposed to ratcheting following spontaneous unfolding [126].

The missing component is almost certainly the action of SecA on polypeptide segments before they reach the SecY channel. In its ATP-bound state, when the SecY channel is open (figure 3*d*(iii,iv)), the SecA clamp (figure 2*b*) grips tightly onto a segment of pre-protein. This is evidenced by slow pre-protein dissociation rates [81] and the protection of this region of SecA from HDX [104]. This ATP-promoted interaction would prevent backsliding of protein that has already entered the central chamber between SecA and SecYEG (downstream of the ‘clamp’ in figure 3*d*(iii)). However, it would equally prevent the next stretch of pre-protein from diffusing into this space. Closure of the clamp may also be capable of unfolding structured elements as they enter SecA, thus ensuring that pre-protein is translocation-competent as it approaches the membrane channel.

Meanwhile, in the ADP-bound state (figure 3*d*(i,ii,iv)) the reverse seems to be true: pre-proteins can more freely enter the cytoplasmic cavity (larger dotted arrow in state i), but only less positive or non-bulky sequences are able to pass through SecY to the periplasm unhindered. Furthermore, SecA bound to ADP has a higher propensity for dissociation [81,92], allowing backsliding as far as the most recent blockage in the periplasm (figure 3*d*(v), dotted arrow), or potentially even further [122,128].

For this mechanism to drive transport, sections of up to 5 kDa pre-protein (for 1 STEP) must readily accumulate within the translocon, after the SecA clamp but before the SecYEG channel (‘cavity’ in figure 3*d*(i); filled by transition to ii). The original Brownian ratchet model posited that this would be relatively energetically neutral, with these sections entering randomly and triggering nucleotide exchange once they have entered the cavity—and thus biasing the direction of diffusion. However, it could be that this movement is intrinsically energetically favourable, with ADP-induced conformational changes creating a structured space into which pre-protein naturally flows. A third possible scenario is that this accumulation in the central cavity is unfavourable, but driven by the ATPase cycle of SecA in a more tightly coupled manner—perhaps even a single amino acid at a time, as in the mechanism proposed on the basis of the recent structures (outlined above, figure 3*b*). This latter situation might be expected on entropic grounds: if energy is used to push polypeptide into a confined space (states (i) → (ii) in figure 3*d*), then the open channel induced by ATP would provide a ‘release valve’, adding a further forwards driving force by promoting the (iii) → (iv) transition. Distinguishing these possibilities will require single molecule reporting of polypeptide movement through the channel alongside each stage of the ATP hydrolytic cycle.

Crucially, the plausible possibilities described above all still rely on Brownian motion for the passage of polypeptide across the membrane: the action of SecA may cause stretches of unfolded (translocation-ready) pre-protein to accumulate at the entrance to SecY, but the final, rate-determining step for the entire process is the diffusion of polypeptide through the channel; more specifically, the diffusion of positively charged residues (arginines; see below for lysines) and to a lesser degree bulky residues within them. Auxiliary transport factors (see below) can thus promote transport at the point of diffusion ((iii) → (iv) in figure 3*d*), either by promoting forward movement or by inhibiting the reverse.

## 11. The role of the PMF

It is well known that the presence of PMF stimulates pre-protein transport directly [8], even in the absence of SecDF (see also below). However, for many years the mechanism for this enhancement was not understood. In this respect, the transport of positively charged amino acids is interesting. Lysines are generally deprotonated for passage through the Sec machinery, while the only other amino acid that is positively charged at physiological pH—arginine—is less common in secreted compared to unsecreted proteins [123]. Therefore, essentially all pre-proteins will have a net negative charge as they transit through SecYEG [123].

This suggests a ‘proton-rachet’ mechanism, which could in theory harness both the electrical (*Δψ*) and chemical (*Δ*pH) components of the PMF to bias the direction of polypeptide diffusion. The former would drive negatively charged residues towards the positively charged periplasm by electrophoresis (figure 3*e*) [129], while the latter would facilitate the neutralization of positive charges on lysines, thereby rendering the translocating chain more electro-negative (figure 3*f*). Additionally, the re-protonation of lysines at the exterior (favoured at the lower pH there) would help prevent backsliding due to SecY's known impermeability to positive charges [115,130] (figure 3*f*). The presence of an electric field across the membrane may also induce conformational changes in the Sec machinery, or otherwise alter the energetics of transitions between states to promote transport towards the periplasm, in ways have yet to be described.

## 12. Other forces and auxiliary components that influence transport

Because transport is fundamentally diffusional, any factor that enhances forward diffusion ((iii) → (iv) in figure 3*d*), or inhibits backsliding ((iv) → (iii)) will contribute. One such factor is intrinsic to the core Sec machinery: modulation of pre-protein folding. Binding of ATP to SecA causes a restructuring of the central cavity, promoting unfolding of pre-protein secondary structure, and thus forward movement [101] (figure 3*g*). Once across the membrane, secondary structure reforms—either spontaneously or facilitated by the exterior surface of the channel (and accessory factors; see below)—and thereby prevents backsliding (figure 3*g*).

A number of auxiliary Sec components are also thought to enhance transport by acting at the (iii) → (iv) diffusion step. The SecDF sub-complex, for example, associates directly with the Sec translocon *in vivo* and stimulates pre-protein transport using energy derived from the PMF [131,132]. A general mechanism for this has been proposed based on structural and functional data, in which the large periplasmic domain of SecDF binds to pre-proteins as they emerge from SecY then pulls them out into the periplasm, somehow harnessing proton flux through the trans-membrane region to provide directionality (figure 3*g*) [133].

Other SecYEG binding partners, such as periplasmic chaperones PpiD, YfgM [134,135], SurA and Skp might also contribute to the transport process, again by associating with the pre-protein emerging through SecY and thereby preventing backsliding (figure 3*g*). Interestingly, a role has been proposed for the Sec interactors PpiD/YfgM in assisting SecDF in the secretion of the *Vibrio* protein VemP [136,137]. Furthermore, we have recently shown that SurA interacts with the periplasmic regions of SecDF, presumably to also help facilitate the latter stages of secretion [138]. The complete SecYEG interactome is fairly large and not always well characterized [134,139], so it is likely that other proteins are also involved in secretion, potentially to help achieve the required rate, but in ways that remain to be elucidated.

Beyond Sec-associated proteins and the PMF, the presence of specific lipids—notably cardiolipin (CL)—are important for ensuring that Sec transport is at its fastest [140–142]. Indeed, CL seems to be important for the conferring PMF-stimulation to transport across the inner membrane [140], perhaps by assisting in the rapid dissipation of protons away from translocating lysines at the cytosolic surface. There is also evidence that the way the transporter complexes are assembled into the membrane can affect their function [118]. Together, all these factors—along with the fact that pre-proteins are presumably delivered to the Sec machinery *in vivo* in an optimally secretion-competent conformation—probably account for the estimated 1–2 orders of magnitude difference in transport rate between the *in vitro* reconstituted core Sec machinery and secretion through the holo-translocon *in vivo*.

## 13. Events after transport

Once most or all of transport has taken place, the signal peptide is removed by signal peptidase to produce the mature protein [143]. Without this step, pre-proteins stay anchored to the inner membrane and are unable to reach their final envelope, or external, destination [144]. Even after SS removal, however, it is likely that pre-proteins remain associated with the periplasmic exit to the channel until they are collected or released by chaperones, which either then help them fold or shepherd them to their next destination. For example, processed β-lactamase has been shown to remain associated with the inner membrane until it has folded [145], while deletion of the chaperone PpiD similarly slows the release of processed OmpA from inner membrane vesicles [146]. *In vitro* at least, this resetting of the channel is often rate-limiting, as turnovers beyond the first are substantially slower or even non-existent *in vitro* [92,116]. Presumably, therefore, it is important to ensure that proteins emerging from the channel are not released blindly into the periplasm; indeed, emerging data suggest that transport through Sec and downstream events such as insertion of OMPs into the outer membrane are much more tightly coupled than was originally thought [11,139,147].

## 14. Conclusion

Protein transport is one of the central functions of a cell, and now for the first time we can reasonably claim to understand how it works—at least in broad strokes and for the core bacterial Sec machinery. The final, unifying model we propose is a combination of all the mechanisms in figure 3*d–g*, with multiple factors coming together to promote transport. This model has arisen from breakthroughs in many different techniques: triumphs of electron microscopy revealing structures of translocon complexes frozen in the act of transporting, but also time-domain information—activity assays and the capture of molecular motions by FRET. These same techniques provide opportunities for the elucidation of the mechanistic basis for the transport of many other metabolite and polymeric clients across biological membranes.

## Data Availability

This article has no additional data.
